# Supplementary material for: Single-Cell Dissection of the Serrated Pathway: Cellular Heterogeneity and Genetic Causality in Colorectal Cancer
Source: Int J Mol Sci. 2025 Jul 25;26(15):7187. doi: 10.3390/ijms26157187 (PMC12347469; doi:10.3390/ijms26157187)
Supplement: Supplementary file 1 [file ijms-26-07187-s001.zip › Figure S2 The TSNE plot shows the big cell type for CRC single cell transcriptome.pdf]

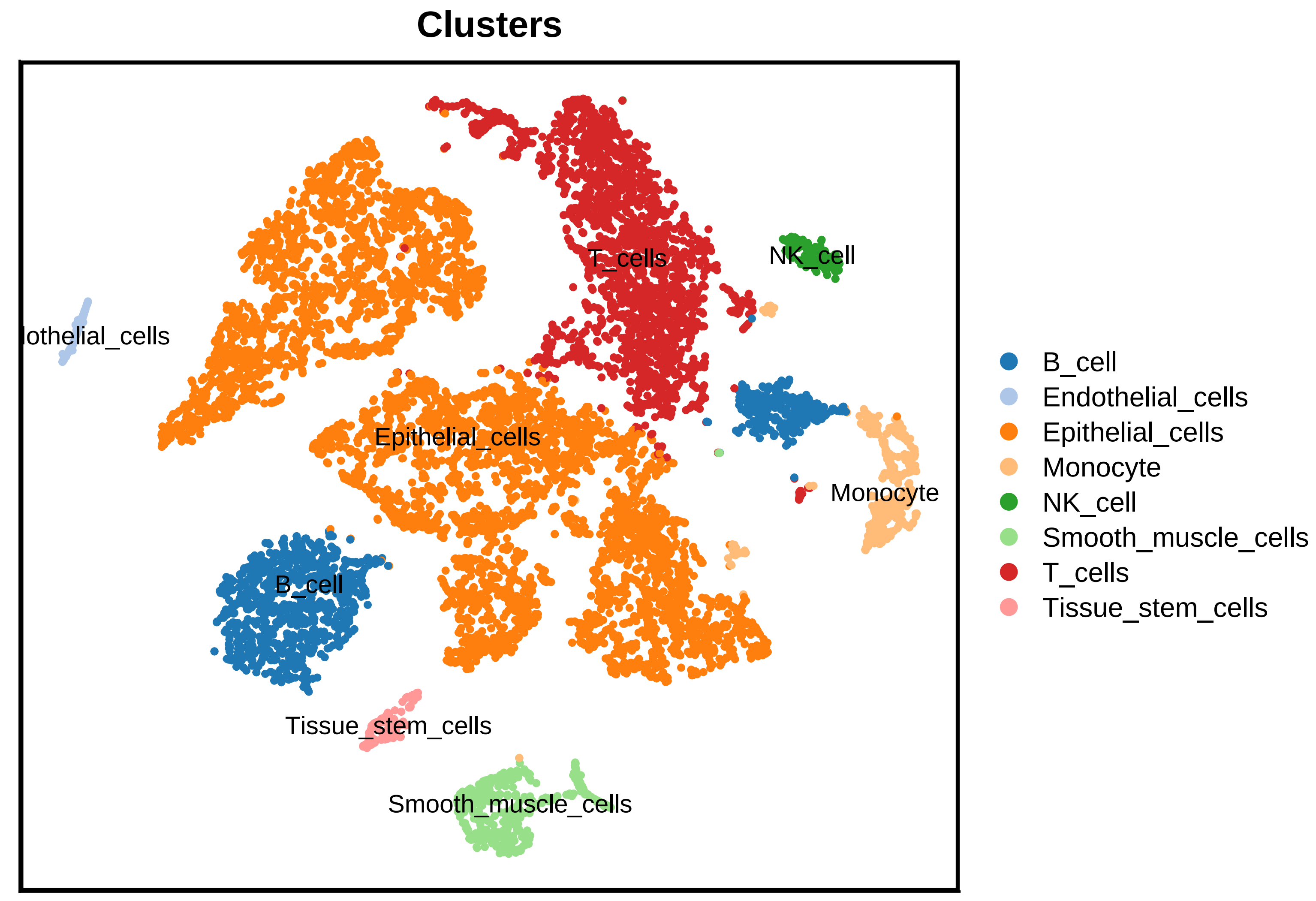
Clusters
lothelial_cells
T_cells
NK_cell
Epithelial_cells
Monocyte
B_cell
Tissue_stem_cells
Smooth_muscle_cells
B_cell
Endothelial_cells
Epithelial_cells
Monocyte
NK_cell
Smooth_muscle_cells
T_cells
Tissue_stem_cells
