# Supplementary figures and images for: Single-Cell Dissection of the Serrated Pathway: Cellular Heterogeneity and Genetic Causality in Colorectal Cancer
Source: Int J Mol Sci. 2025 Jul 25;26(15):7187. doi: 10.3390/ijms26157187 (PMC12347469; doi:10.3390/ijms26157187)

# Clusters

CRC

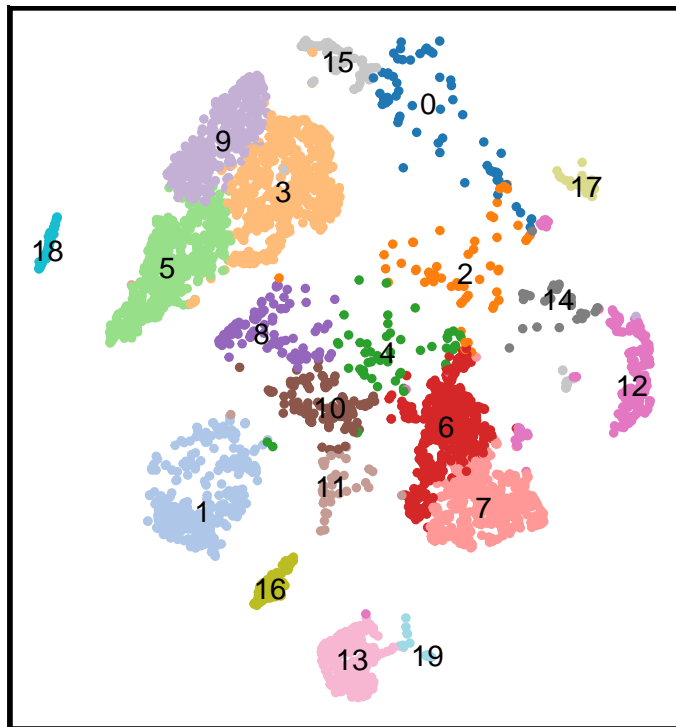

CT

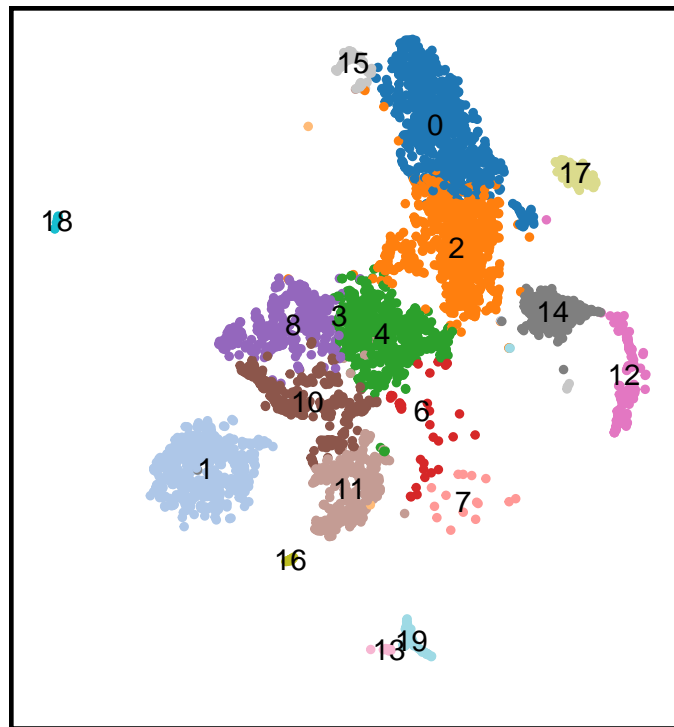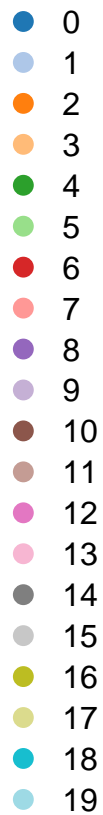

Supplement: Supplementary file 1 [file ijms-26-07187-s001.zip › Figure S1 The cell clusters for CRC single cell transcriptome with resolution of 0.8.pdf]

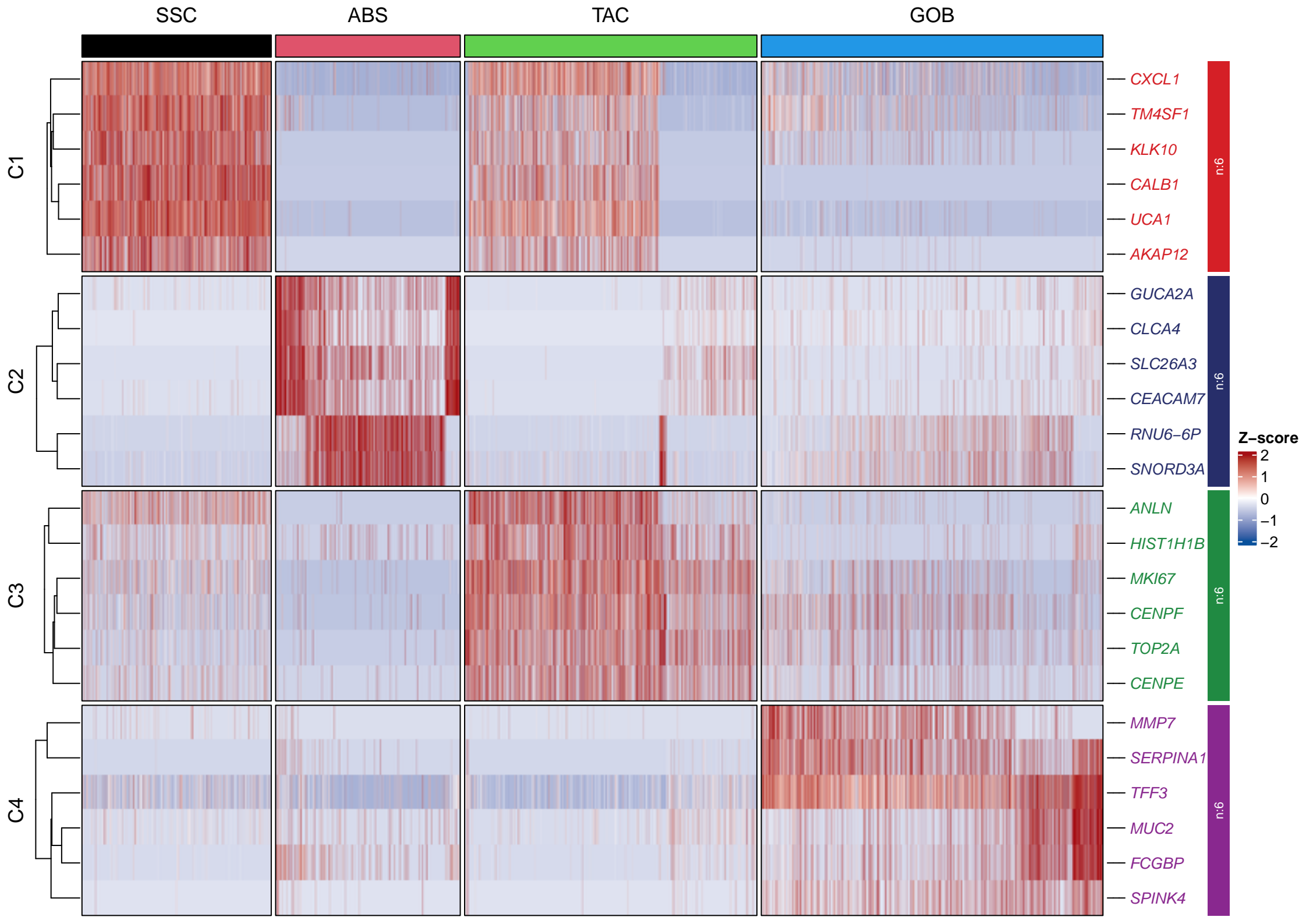

Supplement: Supplementary file 1 [file ijms-26-07187-s001.zip › Figure S3 The marker genes for EPI.pdf]
